# Achieving High-Performance Green Composites from Pineapple Leaf Fiber–Poly(butylene succinate) through Both Fiber Alignment and Matrix Orientation across the Thickness

**DOI:** 10.1021/acsomega.3c02690

**Published:** 2023-09-20

**Authors:** Sorn Duangsuwan, Taweechai Amornsakchai, Pranee Phinyocheep, Sombat Thanawan

**Affiliations:** † Polymer Science and Technology Program, Department of Chemistry, Faculty of Science, Mahidol University, Phuttamonthon 4 Road, Salaya, Nakhon Pathom 73170, Thailand; ‡ Center of Sustainable Energy and Green Materials, Faculty of Science, Mahidol University, Phuttamonthon 4 Road, Salaya, Nakhon Pathom 73170, Thailand; § Rubber Technology Research Center, Faculty of Science, Mahidol University, Phuttamonthon 4 Road, Salaya, Nakhon Pathom 73170, Thailand

## Abstract

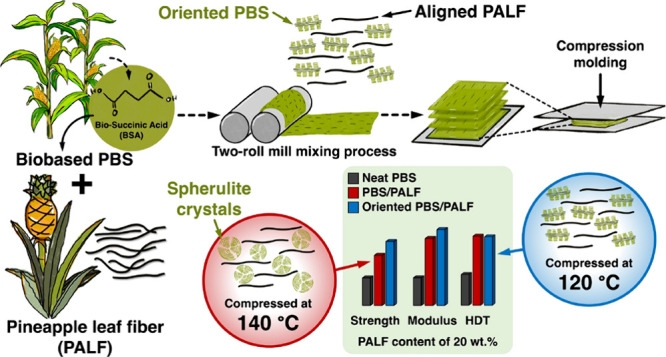

This research aims to develop high-performance and low-carbon composites using biobased poly(butylene succinate) (PBS) reinforced with well-aligned pineapple leaf fibers (PALF). PBS/PALF composites containing 10 and 20% PALF by weight (wt %) were prepared using a two-roll mill. During the mixing process, the molten material was slightly stretched to align the fibers in the machine direction, forming a uniaxial prepreg. The prepreg was subsequently stacked and compressed into composite sheets at compression temperatures of 120 and 140 °C. Differential scanning calorimetry, X-ray diffraction, and crystalline morphology analysis revealed the presence of matrix orientation in the prepreg, which was preserved in sheets compressed at 120 °C but not at 140 °C. The composites prepared at 120 °C exhibited significantly higher flexural strength and modulus compared to those prepared at 140 °C, attributed to the combined effect of matrix and PALF orientation. Additionally, the composites displayed an increase in heat distortion temperature, with a maximum of 10 °C higher than the matrix melting temperature (∼113 °C) for the composite with 20 wt % PALF. These findings indicate the potential for increased utilization of this low-carbon green composite.

## 1 Introduction

Polymer matrix composites are widely used in different industries due to their many advantages. One of that is an automotive industry, which uses polymer matrix composites instead of metals and alloys to reduce the weight of vehicles without compromising vehicle performance or safety.^1^ Lowering the vehicle’s weight would, in turn, reduce fuel consumption, carbon dioxide, and greenhouse gas emissions. However, traditional composite materials are often made from petroleum-based plastic reinforced with either carbon or glass fiber,^2,3^ which are nonrenewable and nonsustainable. In addition, in the production process, carbon dioxide emissions are very high, and this contributes to global warming.^4^ Green composite materials based on biopolymers and natural fibers are being researched and developed to replace traditional composite materials in response to these problems.^5,6^

Interesting biopolymers could be either drop-in polymers such as biopolyethylene and biopolypropylene or new polymers such as poly(lactic acid) (PLA) and poly(butylene succinate) (PBS). The latter receives much greater attention due to the claim of biodegradability, which is, in fact, still controversial. PLA is known not to degrade easily in the environment but will require industrial composting facilities. PBS, on the other hand, degrades more readily in the environment.^7−10^ In addition, PBS has more attractive properties, such as excellent flexibility and thermal stability. PBS produced in Thailand is partially biobased but is still not widely used, possibly due to its low mechanical properties. These problems have been researched and overcome with natural fiber reinforcement.^11−14^ Several literature has shown the efficacy of using natural fiber-reinforced fibers in composites for automotive applications.^15−17^ However, most natural fibers often need to be explicitly grown for the fiber. This requires large amounts of water, fertilizer, and space, which affects the environment. Pineapple leaf fiber (PALF) is another interesting reinforcing material. PALF is produced from agricultural waste obtained from pineapple plantations. It has comparable or better mechanical properties compared to other natural fibers.^18,19^ PALF, as a reinforcing fiber, can improve composite mechanical properties and reduces the environmental impact, production costs, and plastic use and helps to store or sequester carbon in the products. This is another effective way to mitigate global warming.^20,21^ In addition to fiber properties, the mechanical properties of the composite also depend on the composite production process. It has been shown that with an injection molding process, composites with much improved mechanical properties may be obtained when compared to compression molding due to fiber alignment and matrix orientation. However, such fiber alignment and matrix orientation usually occur only at the skin.^22−25^ To achieve a thicker alignment zone, it is necessary to use sophisticated techniques such as shear-controlled orientation injection molding (SCORIM) or oscillatory shear injection molding (OSIM).^25−27^ A much simpler process has been proposed and proven to be quite effective.^28,29^ The method does not require a special machine or tooling but only melt mixing and then alignment on a two-roll mill or a similar process to form prepreg. The prepreg can then be consolidated under appropriate conditions at a later stage to form final products. The method can be applied in many composite systems.

Thus, the main objective of this work is to explore the possibility of improving the performance of low-carbon composites derived from PBS by introducing two innovative approaches: fiber alignment and matrix orientation. Notably, in the existing body of research, limited attention has been directed toward the alignment of fiber in such composites. Furthermore, the role of the matrix orientation remains largely unexplored. To address these gaps, this study employs a novel method to prepare thin composite “prepregs” with enhanced fiber alignment, achieved through a laboratory two-roll mill. The matrix orientation within the “prepregs” is manipulated through controlled melt stretching. Subsequently, the “prepregs” undergo compression to form sheets under distinct temperature conditions, i.e., one designed for matrix consolidation and another intended to disrupt any existing molecular structure or morphology within the “prepregs”. The comprehensive investigation encompasses mechanical and thermal properties, correlated with X-ray diffraction data and morphological studies. By introducing these innovative concepts and methodologies, this work seeks to contribute to the advancement of sustainable composites, offering insights into unexplored areas of fiber alignment and matrix orientation, which have the potential to significantly enhance the performance of PBS-based materials for diverse industrial applications.

## 2 Results and Discussion

### 2.1 Proof of PALF Alignment in Composite Prepreg

**Figure 1:**
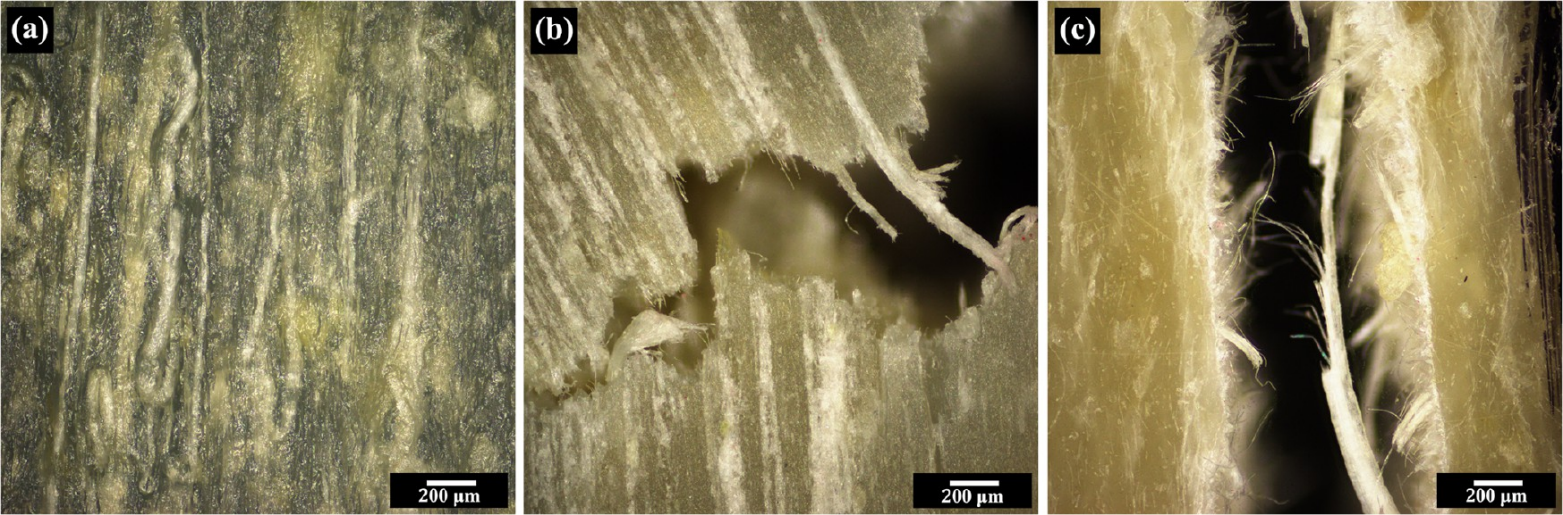
Optical micrograph of (a) PBS/PALF prepreg and fracture surfaces of PBS/PALF sheets in directions perpendicular (b) and parallel (c) to the machine direction. The machine direction is vertical.

Optical micrographs of PBS/PALF composite prepreg and its fractured samples of composite sheets in the direction perpendicular and parallel to the machine direction are shown in Figure 1. PALF is seen to align parallel to the machine direction across the specimen thickness. This evidence indicates that uniaxial PBS/PALF composite prepreg can be successfully prepared by the two-roll mill mixing process and the alignment can be preserved after compression molding.^28,29^ This will be reconfirmed with other techniques in the following section.

### 2.2 Proof of Matrix Orientation in Composite Prepreg and Compressed Sheet

Our intention was to induce a certain level of preferred orientation in the composite prepregs. This was achieved by stretching the molten polymer mixture during prepreg preparation, as described in Experimental Section. If the operation were effective, it would lead to the development of specific internal structures and morphologies. We anticipated the formation of a shish-kebab structure in the prepregs. Therefore, different techniques were employed to ascertain whether such a structure was indeed developed and present in the prepregs.

#### 2.2.1 Differential Scanning Calorimetry (DSC)

**Figure 2:**
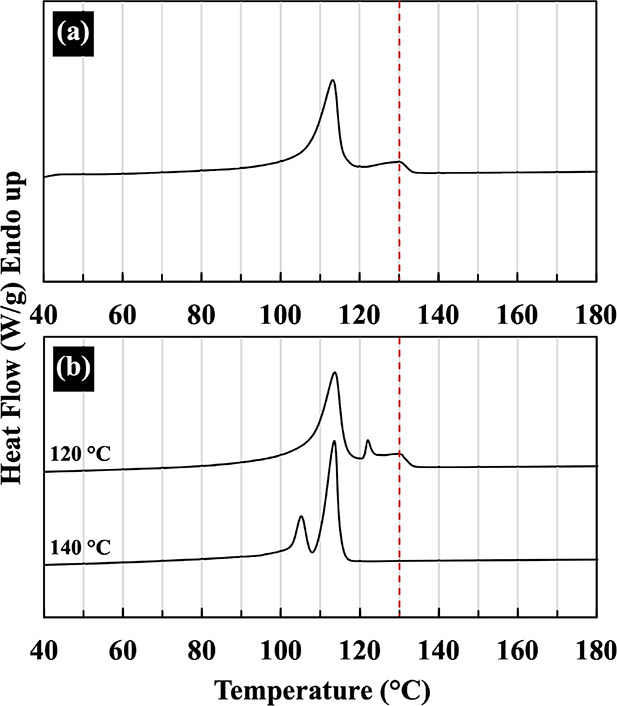
DSC thermograms of (a) first heating curve of PBS prepreg and (b) second heating curve of neat PBS prepreg after being heated to 120 and 140 °C and then cooling. The red dotted line represents *T*_*m*2_ of the composite prepreg for easy comparison.

Figure 2 displays DSC thermograms of starting PBS prepreg and prepregs that had been heated to 120 and 140 °C. The starting prepreg displays two melting temperatures at approximately 113 °C (*T*_*m*1_) and 130 °C (*T*_*m*2_) (Figure 2a), indicating two populations of crystallites. The *T*_*m*1_ region represents the typical melting temperature of PBS crystals (spherulitic structure) commonly observed and reported in the literature.^30,31^ The higher melting temperature (*T*_*m*2_) is associated with a thicker lamellar or chain-extended structure. Therefore, it can be deduced that the PBS prepreg possesses a shish-kebab structure. Similar thermal behavior can be observed in isotactic polypropylene (*i*PP)^32^ and polyethylene^33,34^ with a shish-kebab structure. This observation also confirms that the method use is effective enough to cause nuclei (shish or chain-extended core) to develop from chains aligned under elongation or shear flow. Then, surrounding molecules deposit on the lateral surface of the nucleus and grow as folded-chain lamellar crystals (kebab) along the length of the shish.

The fact that there are two separate melting regions allows us to choose the consolidation temperature that preserves or destroys the initial structure. To test this assumption, prepreg samples were heated in a DSC to a temperature midway between the two melting regions (120 °C) and beyond the higher temperature (140 °C) before cooling back to room temperature. Figure 2b displays the DSC thermogram of the samples, showing that the one heated to 120 °C exhibits a trace similar to that of the starting prepreg, whereas the one heated to 140 °C does not.

**Figure 3:**
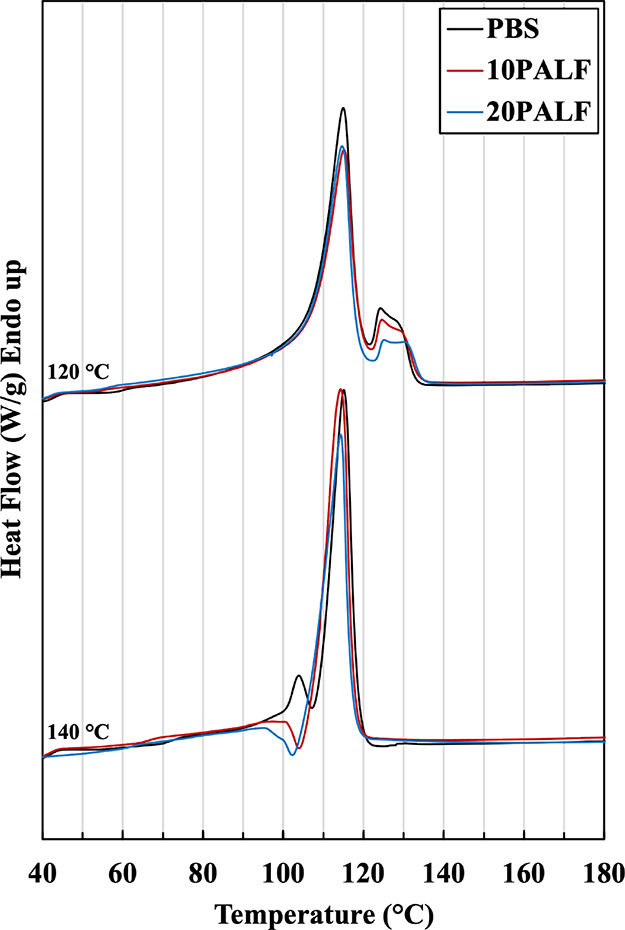
DSC thermograms of neat PBS and PBS/PALF composite sheets containing different PALF contents compressed at 120 and 140 °C.

Similar results were obtained from compressed sheets of PBS and its composites prepared at 120 and 140 °C, as shown in Figure 3. However, the peak height may decrease slightly due to the increased PALF ratio.

#### 2.2.2 X-ray Diffraction (XRD)

**Figure 4:**
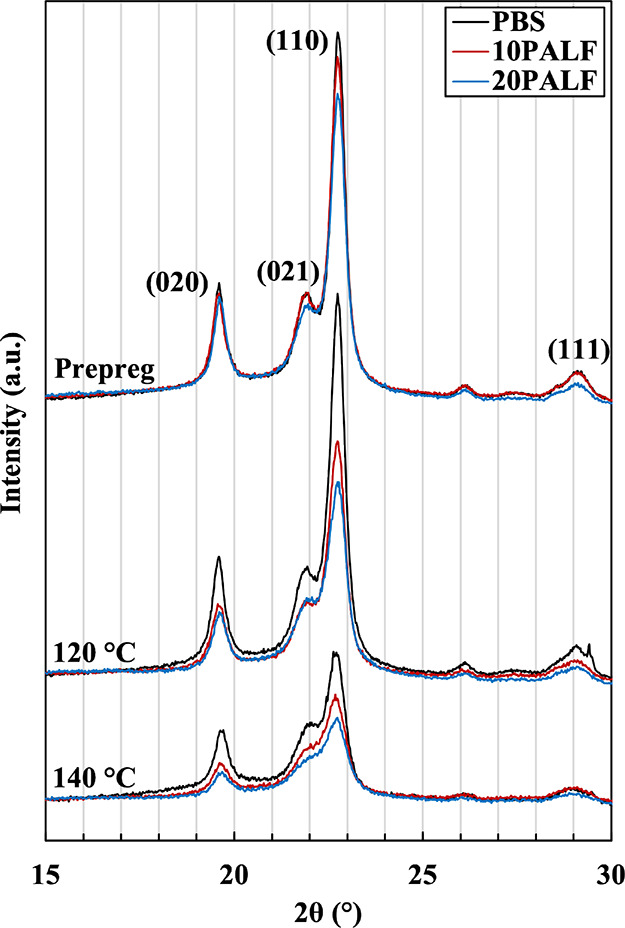
X-ray diffraction patterns of PBS and PBS/PALF composite prepreg and sheet compressed at 120 and 140 °C.

X-ray diffraction patterns of prepreg and sheets compressed at 120 and 140 °C are shown in Figure 4. Peaks appear at 19.6°, 21.9°, 22.7°, and 29.1°, which correspond to (020), (021), (110), and (111) reflections, respectively, of the PBS crystalline phase. These peak positions are similar to those generally observed, except that the intensity of the (110) reflection in our samples is much stronger.^35^ This is due to the preferred orientation in prepregs, as described above. To confirm this, the prepregs were heated to 120 and 140 °C to partially and completely destroy the structure created during the prepreg preparation stage. As the prepregs were heated to 120 °C, the intensity of the (110) reflection decreased slightly and also became broader. The peak intensity dropped significantly when the prepregs were heated to 140 °C. The relative peak intensities for these samples are closer to those generally observed for isotropic PBS.^36,37^ Thus, it can be concluded from the XRD results that all prepregs and composite sheets prepared at 120 °C have a preferred crystalline orientation, while those prepared at 140 °C do not.

#### 2.2.3 Pole Figures

**Figure 5:**
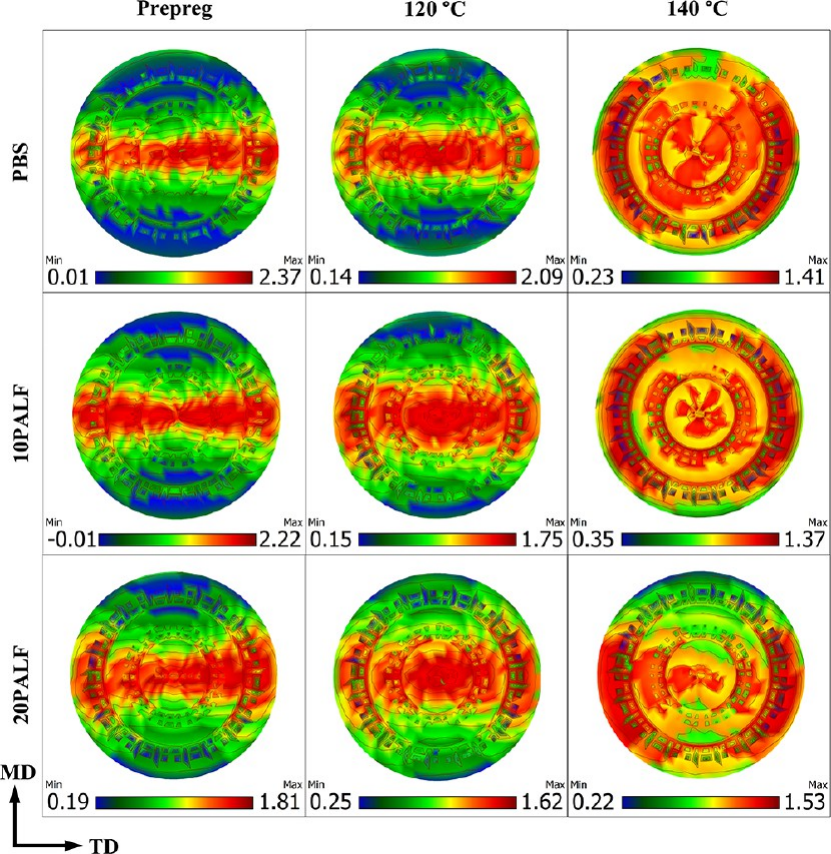
X-ray pole figures for the (110) plane of PBS and PBS/PALF composite prepreg and sheet compressed at 120 and 140 °C. The machine direction (MD) is vertical.

Pole figures for all samples for the (110) plane are listed in Figure 5. It is clearly evident that the peak intensity of the prepreg and composite sheet compressed at 120 °C is concentrated in the center, indicating a preferred matrix orientation in both samples. The presence of a high intensity region supports the fact that (110) reflection of the drawn PBS film lies on the equator.^38^ However, when the sample was compressed at a higher temperature (140 °C), the previous orientation disappeared. These results are consistent with the previous XRD and DSC findings. Notably, with an increased fiber content in the sample compressed at 140 °C, a relatively weak molecular orientation can still be observed. This suggests that the presence of fibers could slow down the relaxation of the matrix in the vicinity of the fiber, as suggested in the literature.^28^

#### 2.2.4 Internal Morphology

**Figure 6:**
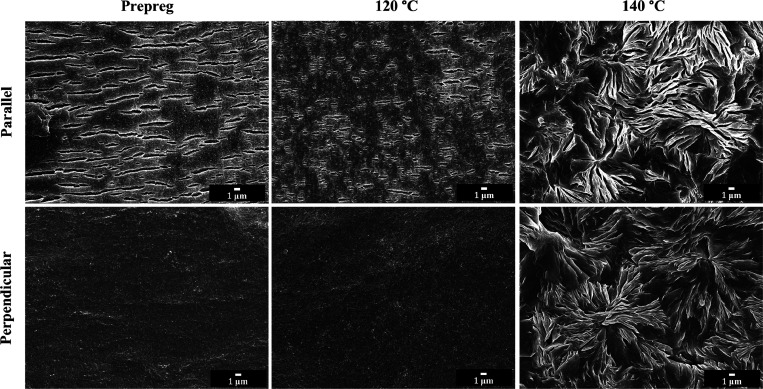
Scanning electron micrographs of etched surfaces of PBS prepreg and sheet compressed at 120 and 140 °C in the directions parallel and perpendicular to the machine direction.

Matrix orientation was also confirmed by observing the internal morphology of PBS prepreg and compressed sheets. Figure 6 displays internal morphologies of the PBS prepreg and sheets compressed at 120 and 140 °C in both parallel and perpendicular directions to the machine direction after etching away the amorphous phase. The PBS prepreg and sheet compressed at 120 °C show very different morphology between parallel and perpendicular directions. The morphology in the parallel direction is composed of many small and long voids, while that in the perpendicular direction is rather featureless. Voids in the sheet compressed at 120 °C are shorter than those seen in the prepreg. These voids are amorphous regions between lamellae, which were removed after etching.^25,39^ The difference in the morphology in the two directions indicates the anisotropic nature of the material. On the other hand, the morphologies in the two directions of composites prepared at 140 °C are very similar in that they have a spherulitic structure and thus are isotropic. This is similar to what is seen in the polypropylene system.^28^

**Figure 7:**
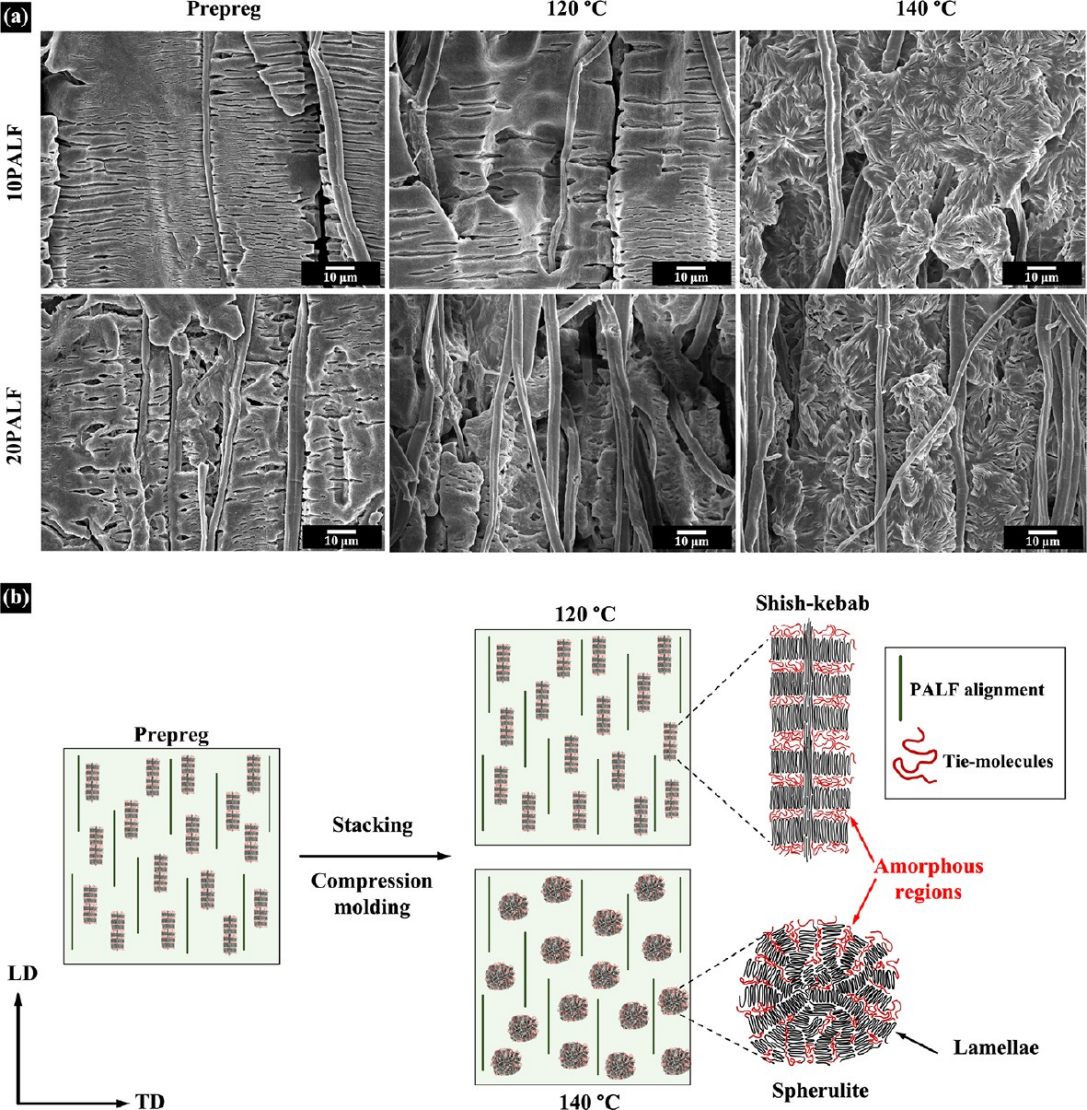
(a) Scanning electron micrographs of etched surfaces of composite prepregs and sheets compressed at 120 and 140 °C in the direction parallel to the machine direction. (b) Illustration of the architecture of shish-kebab and spherulite structures in PBS/PALF composites.

To further demonstrate that the phenomenon described above is also observed in compressed sheets, the internal structure of the composites prepared under similar conditions was investigated. Figure 7a displays the internal morphology of the PBS/PALF composite prepreg and sheets compressed at 120 and 140 °C. The composite prepreg and sheet compressed at 120 °C show voids perpendicular to the machine direction, similar to what was observed in Figure 6 for PBS but more regular. PALF is also seen to align parallel to the machine direction. For the sheet compressed at 140 °C, a spherulitic structure is seen, as discussed above for PBS. For a clearer observation, an illustration of the architecture of the crystalline appearance resulting from the influence of the compression temperature is shown in Figure 7b. The red lines represent the tie molecules or the amorphous regions between lamellae, which could be removed during the etching process. Thus, the prepreg and sheet compressed at 120 °C exhibited elongate voids perpendicular to the machine direction. Therefore, it can be ascertained that the matrix molecules are aligned in the same direction as the machine and structure should be shish-kebab as reported in other systems.^27^ Hence, it may be stated that the presence of PALF does not interfere with the crystallization of PBS as observed in the PBS/kenaf system.^30^

#### 2.2.5 Mechanical Properties

**Figure 8:**
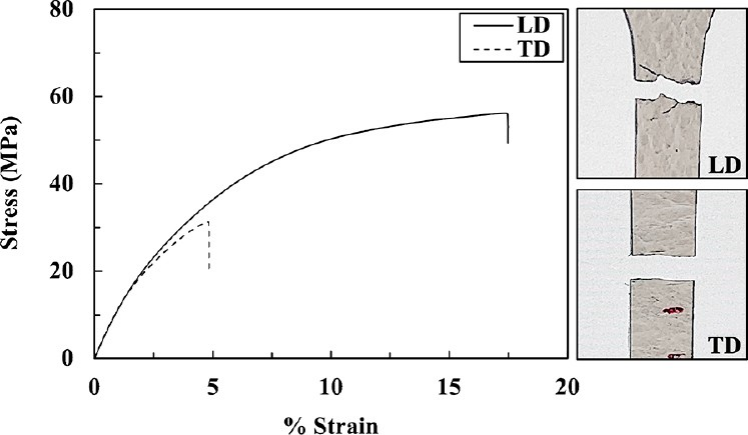
Tensile stress–strain curves and tensile fracture specimen of PBS prepreg in the LD and TD directions. Figures on the right display the fracture characteristics in two directions.

Figure 8 shows the tensile stress–strain curves of the prepreg measured in directions parallel (or longitudinal direction (LD)) and perpendicular (or transverse direction (TD)) to the machine direction. The behavior in the two directions is significantly different, and the fracture characteristics also differ. In the LD direction, the fracture is jagged, while in the TD direction, it is rather straight and smooth. The average tensile modulus, tensile strength, and elongation at break of the PBS prepreg in the two directions are summarized in Table 1. The average tensile strength in LD is about 1.8 times that measured in TD, while elongation at break in the LD is much shorter. Surprisingly, the average tensile moduli in the two directions are not significantly different and the value in LD is even smaller. Despite this fact, it can be deduced that the observed behavior is due to matrix orientation in the prepreg.^40,41^ This result is consistent with the previous confirmation of the matrix orientation.

**Table 1:** Mechanical Properties of PBS Prepreg in Two Directions

**sample**	**longitudinal direction**	**transverse direction**
tensile modulus (MPa)	1130 ± 28	1139 ± 49
tensile strength (MPa)	56.1 ± 2.9	31.8 ± 1.1
elongation at break (%)	20.0 ± 8.0	5.1 ± 0.2

The results presented in this section clearly confirm that the internal structure of the matrix can be controlled by selecting the appropriate consolidation temperature. The initial matrix structure is of the shish-kebab type, which can be preserved by using a low consolidation temperature of about 120 °C. Conversely, an isotropic spherulitic structure is obtained by using a high consolidation temperature of 140 °C to destroy the initial structure and allow the matrix to crystallize in a quiescent state. In the next section, we explore the effects of these controlled structures on the mechanical and thermal properties of the composites.

### 2.3 Effect of Matrix Orientation on Properties of Composite Sheets

#### 2.3.1 Mechanical Properties

**Figure 9:**
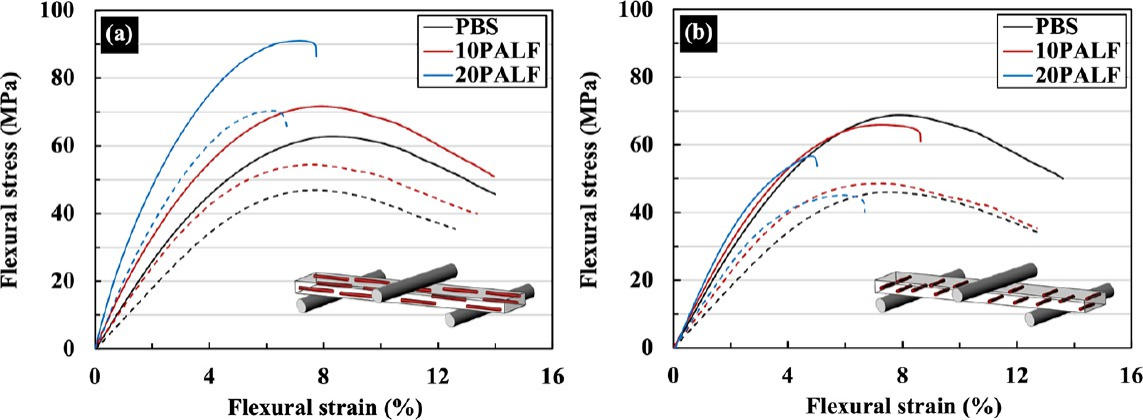
Representative flexural stress–strain curves of neat PBS and PBS/PALF composite sheets measured in two directions: (a) LD and (b) TD. The solid and dashed lines represent the composite sheets compressed at 120 and 140 °C, respectively.

Figure 9 shows the representative flexural stress–strain curves of neat PBS and PBS/PALF composites measured in the LD and TD directions. The slope and maximum stress in the LD increase with increasing PALF content for both types of sheets. In the TD direction, little change in slope is observed, while the maximum stress decreases with increasing PALF content. Additionally, the sheet compressed at 120 °C exhibits a higher slope and maximum stress than the sheet compressed at 140 °C in both LD and TD directions.

**Figure 10:**
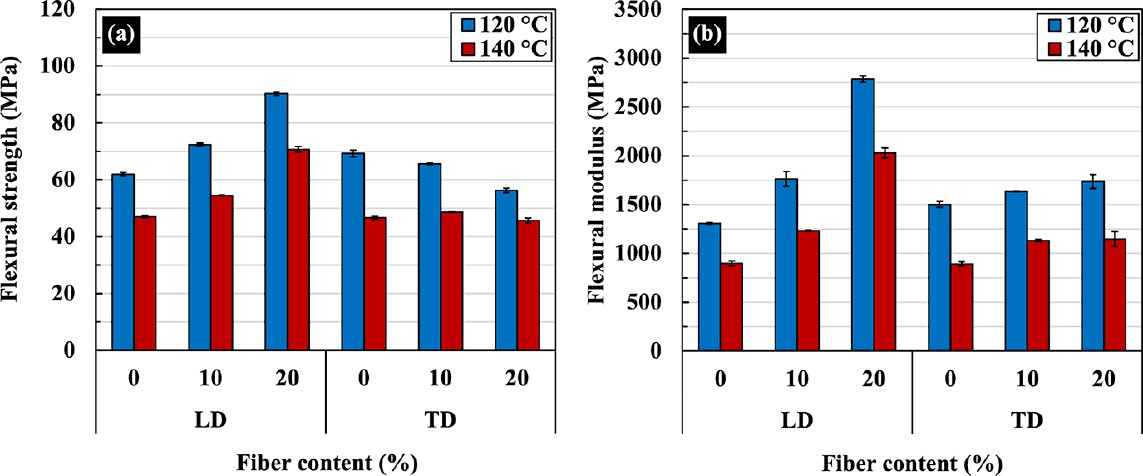
(a) Flexural strength and (b) flexural moduli at 1% strain in two directions of PBS/PALF composite sheets compressed at 120 and 140 °C.

Figure 10 displays average values for flexural strengths and moduli at 1% strain for all materials described above. Properties in the LD will be considered first, and it is seen that both strength and modulus increase with increasing PALF content for sheets compressed at both 120 and 140 °C. Strengths and moduli of sheets compressed at 120 °C are higher than those of sheets compressed at 140 °C. This indicates that the improvement is due to the matrix orientation. When the strength and modulus in the TD are considered, a different pattern of behavior is seen. Surprisingly, the strength of the neat PBS sheet compressed at 120 °C is similar to that measured in the LD despite clear matrix orientation. When PALF was added to 10 and 20 wt %, transverse strength only drops slightly with increasing PALF content. However, transverse strengths of sheets compressed at 140 °C drop from that of 120 °C and appear not to change with the PALF content. All composite materials, including neat PBS, have virtually the same strength. For the modulus, the effect of PALF content remains observable, and the modulus increases with increasing PALF content. Moduli of sheets compressed at 120 °C are higher than those compressed at 140 °C. The difference increases with increasing PALF content. Thus, it could be deduced that there is a certain degree of interaction between matrix orientation (or relaxation) and the presence of PALF. It is postulated that the presence of PALF either promotes more matrix orientation during prepreg preparation or slows the relaxation of the matrix during the compression molding process. If the relative peak intensities of X-ray diffraction patterns in Figure 4 are considered, it is found that the composite sheet with 20 wt % PALF and compressed at 140 °C has a higher ratio of (110) than neat PBS compressed at the same temperature, indicating lesser relaxation. This agrees with the pole figure results presented in Figure 5 and that observed in the polypropylene/PALF system.^28^ In addition, it should be noted that moduli for composite sheets compressed at 140 °C in LD and TD are not too different, and the difference increases as the PALF content is increased. Thus, it may be concluded at this stage that matrix orientation within the composite improves both flexural strength and modulus in both directions and that the level of anisotropy, although existing, is not as high as would be expected. The degree of anisotropy for strength is greater than that for modulus. Only when PALF content reaches 20 wt % does the flexural modulus in LD become higher than that in TD. This behavior is unlike other systems, which have a relatively high degree of anisotropy for both strength and modulus.^42,43^

**Figure 11:**
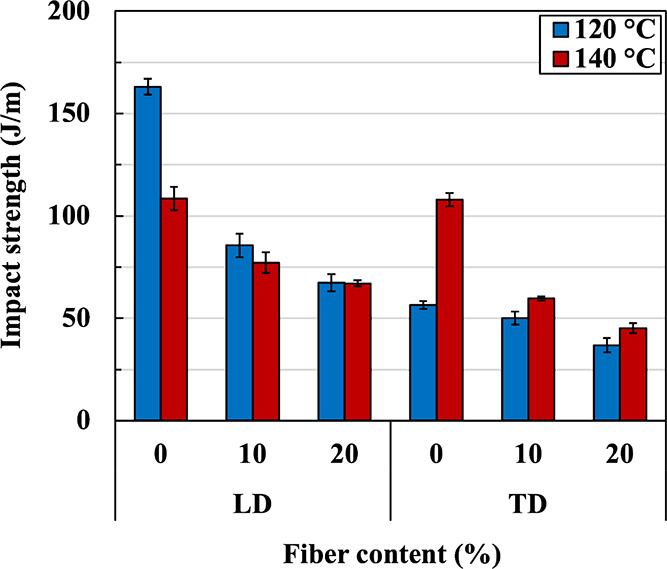
Impact strengths of neat PBS and PBS/PALF composite sheets compressed at 120 and 140 °C measured in two directions.

Impact strength will now be considered, and the results are shown in Figure 11. The effect of matrix orientation can be clearly seen in neat PBS. The impact strength of neat PBS sheet prepared at 120 °C in LD (crack running perpendicular to the machine direction) is much higher than that in TD. When the sample was prepared at 140 °C, the impact strength dropped from that prepared at 120 °C, and no anisotropy was seen, i.e., impact strengths in both directions are virtually equal. For PBS/PALF composites, impact strengths in both directions decrease with increasing PALF content and matrix orientation has little effect. The reduction in impact strength is similar to that reported in other systems.^44,45^

To demonstrate the reinforcement efficiency of PALF in PBS and the composite preparation method used in this work, the results are compared with those of other natural fibers in PBS systems prepared using different techniques found in the literature. Table 2 compares the mechanical properties (flexural properties) of PBS composites reinforced with various types of natural fibers at 20 wt %, except for nanocrystal cellulose (CNC) and kenaf fiber, which are present at approximately 3 and 30 wt %, respectively. The %increment in flexural strength and modulus of this work, as shown in the table, was computed between the most outstanding PBS/PALF composite (20PALF compressed at 120 °C) and the neat PBS (PBS compressed at 140 °C). It was found that the %increment in both strength and modulus was significantly higher than that of most systems with added fibers, such as jute, coir, oil palm, and curaua fibers, all at the same fiber content, including CNC. The good alignment of fibers and matrix may be the main factor contributing to this result. However, even with the matrix orientation factor removed, the %increment in strength and modulus (50 and 126%, respectively) in this work was still significantly higher. Additionally, this could also be attributed to the relatively longer PALF fiber length compared to jute and oil palm composites. Fiber length is another crucial factor influencing the enhancement of mechanical properties that cannot be ignored. The effect of fiber length on composites was clearly demonstrated in kenaf-added PBS. Despite adding up to 30% of fiber content, the strength decreased, and the percentage increment in modulus was not different from that achieved in this work using only 20 wt % PALF. Interestingly, the percentage increment in the mechanical properties, particularly the modulus obtained, was comparable to that of the injection-molded samples, as observed in ramie, PALF/chopstick, and wood composites. Injection molding generally results in better fiber alignment and matrix orientation, thus leading to higher mechanical properties compared to compression-molded samples.^46^ While the systems differ, these results are consistent with those reported in the literature.^47^ It was demonstrated that with proper mixing to prevent fiber breakage and molding conditions to maintain fiber alignment, PALF could provide a higher reinforcement efficiency than aramid fiber in rubber composite systems.

**Table 2:** Mechanical Properties of PBS Composites Reinforced with Different Types of Natural Fibers^a^^,^^b^

		**flexural strength**			**flexural modulus**			
**fiber**	**preparation method (mixing/molding)**	**matrix (MPa)**	**composite (MPa)**	**increment (%)**	**matrix (MPa)**	**composite (MPa)**	**increment (%)**	**ref.**
PALF	TRM/CM	47.0	90.3	92	897	2787	211	this work
jute	ITM/CM	29.8	34.5	16	519	979	88	(12)
ramie	TSE/IM	22.6	39.4	74	423	1365	222	(13)
coir	ITM/CM	40.5	46.2	14	742	1128	52	(14)
oil palm	ITM/CM	37.6	37.6	0	566	781	38	(44)
curaua	dry blending/CM	45.6	57.9	27	1022	2004	96	(48)
CNC	ITM/CM	30.1	31.7	5	473	584	23	(49)
PALF/chopstick	ITM/IM	51.7	75.9	47	880	1700	93	(50)
wood	TSE/IM	37.2	53.6	44	597	1310	119	(51)
kenaf	TSE/CM	40.5	37.3	–8	580	1310	126	(52)

a Mixing: TRM, two-roll mill; ITM: internal mixer; TSE: twin screw extruder. Molding: CM, compression molding; IM: injection molding.

b Data from the literature were estimated from graphs therein.

#### 2.3.2 Fracture Surface

**Figure 12:**
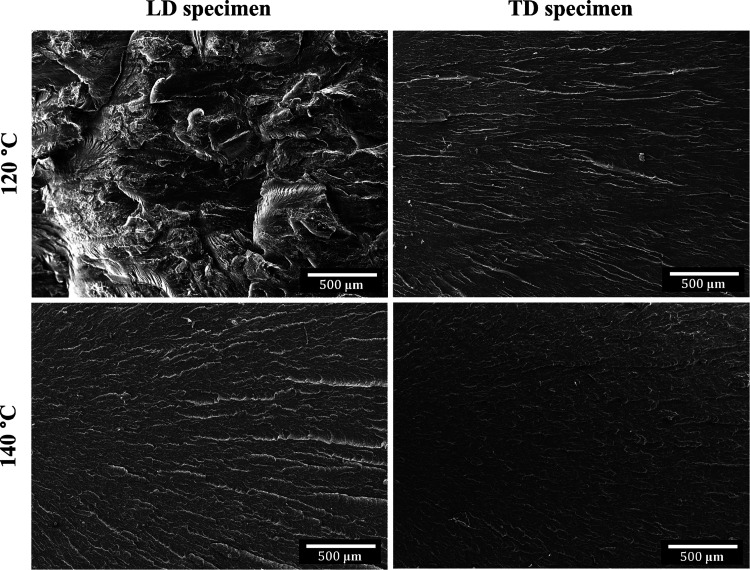
Impact fracture surfaces of PBS compressed at 120 and 140 °C after testing in the LD and TD.

Figure 12 shows the impact fracture surfaces of neat PBS sheets prepared at 120 and 140 °C, which were tested in two directions. The sheet compressed at 120 °C displays very different fracture surface characteristics in the two directions. A rough surface is seen for the LD, while a very smooth surface is seen for the TD. The sheet compressed at 140 °C displays a very smooth fracture surface in both directions. The rough surface relates to the high impact strength, while the smooth surface relates to the low impact strength, and this confirms the previously reported influence of matrix orientation.

**Figure 13:**
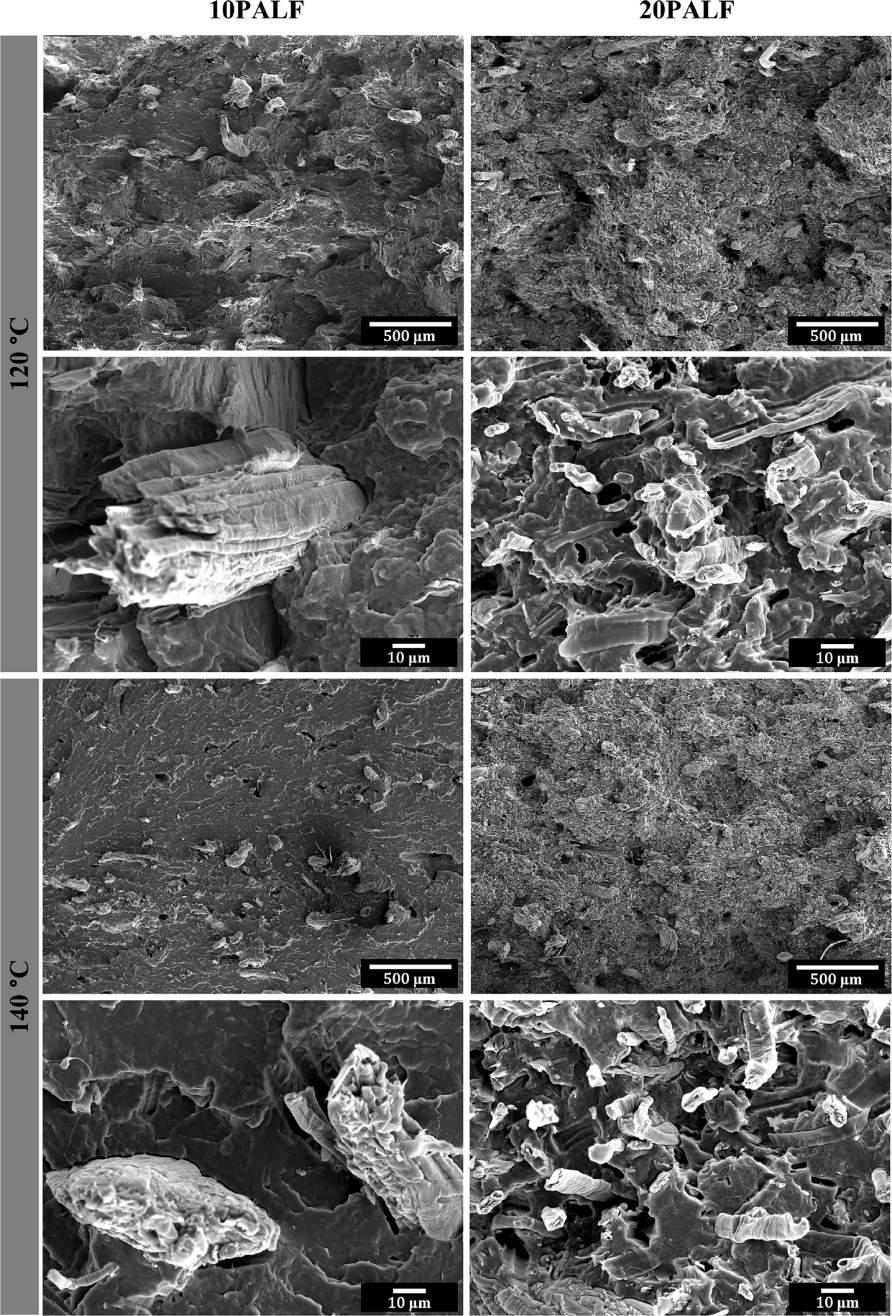
Impact fracture surfaces of PBS/PALF composites containing different PALF contents compressed at 120 and 140 °C in LD. The scale bar for the top row of each temperature is 500 μm, and that for the bottom row is 10 μm.

Figure 13 displays the surfaces of impact-fractured composite specimens that were tested in the LD. For this test, crack propagates in the plane perpendicular to the machine (and fiber) direction. In the composites, fractured fiber’s ends are clearly seen in all composites, indicating that the fibers have good alignment along the machine direction. One point to note is that many large fiber bundles are seen in the composite with 10 wt % PALF, while a few is seen in the composite with 20 wt % PALF. This fact suggests that large fiber bundles break up into small elementary fiber at high PALF content. It is not clear at this moment if this is beneficial to any properties. However, it should be noted that the reduction in impact strength of 20 wt % PALF is not as much as that for the reduction in the first 10 wt % and this could be related to the fact that large PALF bundles in the composite with 10 wt % broke up into many finer elementary fibers.

#### 2.3.3 Heat Distortion Temperature (HDT)

**Figure 14:**
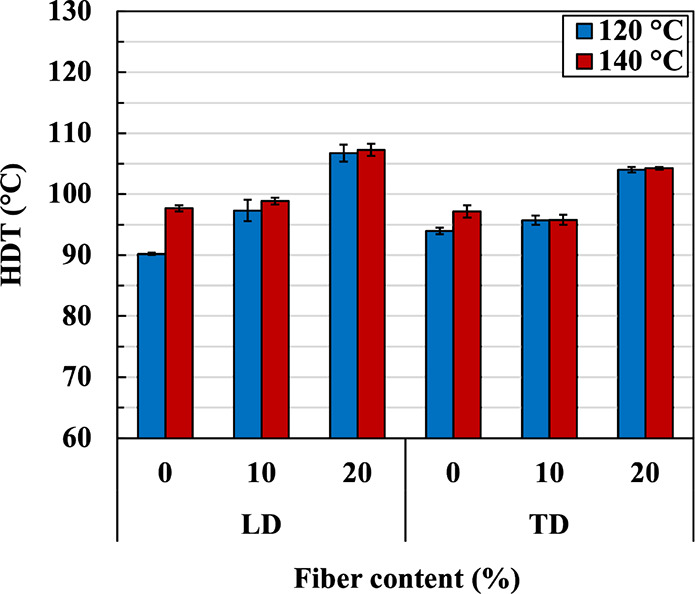
HDT in two directions of neat PBS and PBS/PALF composite sheets containing different PALF contents compressed at 120 and 140 °C.

HDT is one of the important properties that determines whether or not a composite can be used in a certain application such as the automotive industry. Figure 14 shows the HDTs of neat PBS and PBS/PALF composites. The results show that the HDT of sheets compressed at both temperatures increased with increasing PALF content. HDTs in LD are slightly higher than those in TD. The HDT of both composites increased to about 107 °C with 20 wt % PALF, which is very close to the melting temperature of neat PBS at about 113 °C. The effect is much greater than that of grape pomace, which is of rod and particulate shape.^31^ This is due to the PALF contribution to the deformation resistance at high temperatures. This observed behavior is consistent with published results.^21,51,53^ However, the HDT of the composite compressed at 140 °C appears to be slightly higher than 120 °C; this is presumably due to relaxation of the oriented matrix or crystals (in the 120 °C sample) to a random coil state during the long period of the test. This relaxation is similar to what happened with the matrix in the compression process at high temperature. For the sample compressed at 140 °C, the matrix is completely melted, fully relaxed, and crystallized in the compression molding and cooling process. Therefore, the matrix should be dimensionally stable to at least 140 °C and should not change much during the long period of the test. It can also be observed that the difference in HDT values (in LD) of the samples compressed at 120 and 140 °C diminished at high fiber content. This is presumably due to the presence of fibers that slow down the relaxation of matrix molecules, as has been shown in the XRD section. It is worth noting that the results shown here are promising. The high HDT and impact strength of the 20 wt % PALF composites (∼107 °C and 6.6 kJ/m^2^) are comparable to those of the commercial green-composite BioMat (NBF2 112) (∼104 °C and 7.2 kJ/m^2^), which contains 25 wt % hemp fiber, used in automotive interior parts.^54^ This suggests that the composite developed in this study is efficient and could potentially be used as a structural part of automotive interiors.

To summarize, the present study successfully achieved the production of uniaxial PBS/PALF composite sheets through a compression technique, allowing for precise control over PALF and matrix orientation. The observed anisotropic mechanical properties revealed that flexural properties and HDT improved with increasing PALF content, while the impact strength slightly decreased. Furthermore, the choice of compression temperature played a significant role in shaping the composite’s characteristics. By the use of a low compression temperature (120 °C), the matrix orientation was preserved. The presence of PALF slows the relaxation of the matrix during compression molding, and this combined orientation results in superior flexural and impact properties and energy-saving benefits during production. Conversely, employing a high compression temperature (140 °C) disrupted the initial matrix orientation, leading to an isotropic matrix and modified composite properties. The technique’s simplicity also allows for greater flexibility in manufacturing parts, with the thickness easily adjustable by increasing the number of prepregs in the stack. Overall, the PBS/PALF composite demonstrates considerable promise as an eco-friendly alternative, presenting intriguing opportunities for various industrial applications. Notably, the synergy between well-aligned PALF and the molecular orientation of the PBS matrix results in a composite displaying significantly superior mechanical properties when compared to other PBS-based composites, as indicated in Table 2.

In this research, our objective was to formulate and characterize composites based on poly(butylene succinate) (PBS)-incorporating pineapple leaf fiber (PALF), emphasizing the environmentally beneficial aspects of utilizing a biobased biodegradable polymer matrix alongside agricultural waste-derived fiber. While terms like “green” and “low-carbon” were employed to underscore our eco-friendly approach, it is crucial to acknowledge that achieving absolute “greenness” in every process component may not always be feasible; rather, the aim is to prioritize environmentally friendly practices and components while minimizing adverse impacts. A process can retain its “green” designation when its majority of components and practices align with environmental-friendliness, even if some elements fall short. Our focus is on continuous enhancement, striving to minimize the process’s ecological footprint to the fullest extent possible, as exemplified in this study, contributing to the pursuit of sustainable materials with favorable attributes for potential industrial applications.

## 3 Conclusions

Uniaxial PBS/PALF composite sheets were successfully prepared via compression of PBS/PALF prepreg. The technique allows the orientation of both PALF and polymer matrix to be controlled, resulting in composites with improved flexural properties and HDT at higher PALF content, while the impact strength was slightly decreased. The choice of compression temperature is a crucial factor in shaping the composite’s characteristics. By the use of a low compression temperature (120 °C), the matrix orientation can be maintained, leading to composites with superior mechanical properties and energy-saving benefits during production. Conversely, using a high compression temperature of 140 °C destroys the initial matrix orientation within prepreg, resulting in composites with modified properties. This highlights the potential of this eco-friendly composite for various industrial applications, where performance can be tailored to suit specific requirements. The study contributes valuable insights into the field of natural fiber-reinforced polymer composites and encourages further advancements and applications of PBS-based materials.

## 4 Experimental Section

### 4.1 Materials

Poly(butylene succinate) (PBS, BioPBS FZ91PM/FZ91PB) was used as the polymer matrix, produced from the polymerization of biobased succinic acid and 1,4-butanediol. The material was supplied by PTT MCC Biochem Company Limited, and it has a density of 1.26 g/cm^3^ and a melt index of 5 g/10 min (190 °C, 2.16 kg).^7,10^

Short pineapple leaf fiber (PALF) was prepared following the procedure presented in the literature.^18^ Fresh pineapple leaves were collected from cultivation areas in Bang Yang District, Phitsanulok Province, Thailand. After washing, the leaves were chopped into the 6 mm lengths, ground into paste, dried, and sieved to separate out the fibers. The average PALF length is approximately 6 mm, with a diameter ranging from 3 to 68 μm and an average diameter of approximately 20 μm.

### 4.2 Mixing and Prepreg Preparation

Prior to the melt mixing process, both PBS and PALF were dried overnight in a hot air oven at 80 °C. The PBS pellets were then heated and melted on a two-roll mill (W100T, Dr. Collin GmbH, Germany) for 2 min at a speed of 30 rpm. The front and back roll temperatures were 125 and 100 °C, respectively. Subsequently, a predetermined amount of PALF (10 and 20 wt % of total weight (PBS + PALF)) was gradually added over a period of 3 min. The mixing speed was then increased to 48 rpm, and the mixing continued for another 10 min to achieve a homogeneous molten mixture.

**Figure 15:**
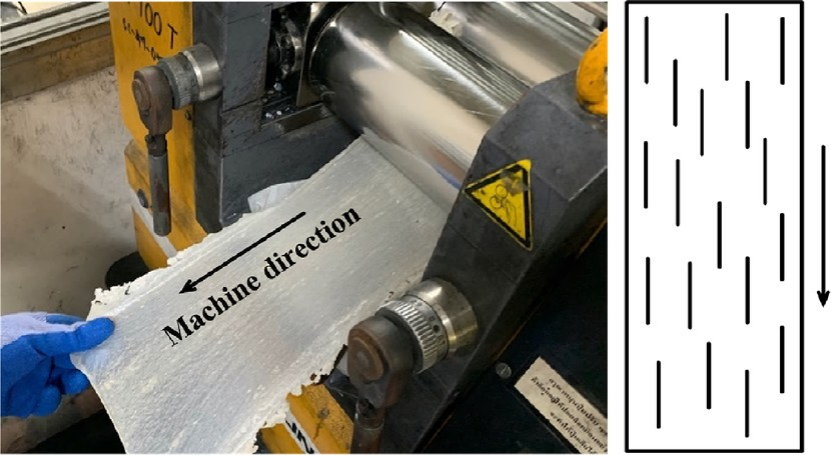
PALF alignment on a two-roll mill during the uniaxial composite prepreg preparation. The schematic on the right shows PALF alignment as represented by black lines.

The resulting molten mixture was carefully pulled out with slight stretching to maintain the alignment of PALF parallel to the machine direction. It was then allowed to cool and solidify, forming prepreg, as illustrated in Figure 15. The composites were designated as 10PALF and 20PALF, denoting the respective content of PALF in the composites.

### 4.3 Compressed Sheet Preparation

Composite sheets were prepared by stacking 10 layers of prepreg between two flat metal sheets and a 3 mm spacer, keeping the fiber aligned in the same direction. The stacked prepregs were preheated for 5 min under slight pressure. Then, it was pressed under a pressure of 1500 psi for 5 min, followed by cooling under the same pressure for 5 min. The compression molding was carried out at temperatures of 120 and 140 °C.

### 4.4 Characterizations

#### 4.4.1 X-ray Diffraction

X-ray diffraction patterns of the PBS/PALF composite containing different PALF contents and compression temperatures were recorded using an X-ray diffractometer (Bruker D8 DISCOVER) over the 2θ range between 5° and 40° with a step size of 0.02°. The X-ray wavelength was 1.54 Å (Ni-filtered CuKα). Pole figures for different samples were obtained with a cradle sample stage on the same machine. The data were analyzed with DEFFRAC.TEXTURE software.

#### 4.4.2 Morphology

##### 4.4.2.1 PALF Alignment

To observe the fiber alignment, the composite prepreg and specimens after the flexural test were observed under an optical microscope (Olympus, BX53M).

##### 4.4.2.2 Crystalline Morphology

The internal morphology of the composites was observed using field emission scanning electron microscopy (FE-SEM) (JEOL InTouchScope, JSM-7610FPlus Schottky) with an accelerating voltage of 10 kV. The samples were etched to remove the amorphous phase using a 0.1 M sodium hydroxide solution in a 1:1 (by volume) water–methanol mixture, which revealed the crystalline morphology.^25^ The etching process was carried out at room temperature for 96 h to ensure complete removal of the amorphous phase. Subsequently, the samples were thoroughly cleaned with distilled water and subjected to an ultrasonic treatment. Prior to observation, a thin layer of platinum was coated with the samples.

##### 4.4.2.3 Fracture Surface

The impact fracture surface morphologies of the composites in LD after impact test were observed with scanning electron microscopy (SEM) (JEOL InTouchScope, JSM-IT500) with an accelerating voltage of 10 kV. Before observation, a thin layer of platinum was coated on the samples.

#### 4.4.3 Thermal Properties

The melting and crystallization behaviors of the composites were determined with a differential scanning calorimeter (TA Instruments, Q200-RCS90). The heating and cooling rate was 10 °C/min under a nitrogen atmosphere.

In addition, the heat deflection temperature (HDT) was determined with a Gotech testing machine (HV-3000-P3C). The specimen size was 120 × 13 × 3 mm^3^. The test was performed following ASTM-D648 under the three-point bending mode with a span of 100 mm under a constant load of 0.455 MPa and a heating rate of 2 °C/min. HDT was determined as the temperature at which the specimen bent to 0.25 mm.

#### 4.4.4 Mechanical Properties

##### 4.4.4.1 Tensile Testing

Tensile testing was carried out on a universal testing machine (Instron 5566) at a crosshead speed of 5 mm/min with 1 kN load cell. The specimens were cut from 0.3 mm-thick prepreg with a dumbbell cutter (ASTM D412 Type C) with a long axis parallel (longitudinal direction, LD) and perpendicular (transverse direction, TD) to the machine direction. The average values of secant modulus at 1% strain and tensile strength at yield from 5 specimens were reported.

##### 4.4.4.2 Flexural Testing

The test was carried out on a universal testing machine (Instron 5569) at a crosshead speed of 5 mm/min, 1 kN load cell, and a support span length of 48 mm. The specimens were cut from compressed sheets into 12.7 mm-wide strips with a long axis parallel (or LD) and perpendicular (or TD) to the machine direction. The average values of flexural strength and secant modulus at 1% strain from 5 specimens were reported.

##### 4.4.4.3 Impact Testing

The test was carried out on a pendulum impact testing machine (Zwick, 2005) in the Izod configuration. The impact specimens were cut from compressed sheets into 60 mm-long and 12.7 mm-wide strips. The samples were notched with a Zwick/Roell manual notch cutting machine. The notches were cut across the LD and TD. The average values of 5 specimens were reported.

## Data Availability

All data generated or analyzed during this study are included in this published article.
